# Can minimally invasive puncture and drainage for hypertensive spontaneous Basal Ganglia intracerebral hemorrhage improve patient outcome: a prospective non-randomized comparative study

**DOI:** 10.1186/2054-9369-1-10

**Published:** 2014-06-01

**Authors:** Guo-Qiang Wang, Shi-Qiang Li, Yong-Hua Huang, Wei-Wei Zhang, Wen-Wei Ruan, Jia-Zhen Qin, Ying Li, Wei-Min Yin, Yun-Jun Li, Zheng-Jun Ren, Ji-Qiang Zhu, Yun-Yan Ding, Jun-Qi Peng, Pei-Jian Li

**Affiliations:** Department of Neurology, General Hospital of Beijing Command, Beijing, 100700 China; Department of Neurology, Xianghe Hospital of Traditional Chinese Medicine, Xianghe County, Hebei Province 654000 China; Department of Neurosurgery, General Hospital of Beijing Command, Beijing, 100700 China

**Keywords:** Intracerebral hemorrhage, Intraventricular hemorrhage, Minimal invasive puncture, Decompressive craniectomy, Recombinant tissue plasminogen activator, Urokinase

## Abstract

**Background:**

The treatment of hypertensive spontaneous intracranial hemorrhage (ICH) is still controversial. The purpose of the present study was to investigate whether minimally invasive puncture and drainage (MIPD) could improve patient outcome compared with decompressive craniectomy (DC).

**Methods:**

Consecutive patients with ICH (≧30 mL in basal ganglia within 24 hours of ictus) were non-randomly assigned to receive MIPD (group A) or DC (group B) hematoma evacuation. The primary outcome was death at 30 days after onset. Functional independence was assessed at 1 year using the Glasgow Outcome Scale.

**Results:**

A total of 198 patients met the per protocol analysis (84 in group A and 114 in group B). The initial Glasgow Coma Scale (GCS) score was 8.1 ± 3.4 and the National Institutes of Health Stroke Scale (NIHSS) score was 20.8 ± 5.3. The mean hematoma volume (HV) was 56.7 ± 23.0 mL, and there was extended intraventricular hemorrhage (IVH) in 134 patients. There were no significant intergroup differences in the above baseline data, except group A had a higher mean age than that of group B (59.4 ± 14.5 vs. 55.3 ± 11.1 years, *P* = 0.025).

The cumulative mortalities at 30 days and 1 year were 32.3% and 43.4%, respectively, and there were no significant differences between groups A and B. However, the mortality for patients ≦60 years, NIHSS < 15 or HV≦60 mL was significantly lower in group A than that in group B (all *P* < 0.05). The cumulative functional independence at 1 year was 26.8%, and the difference between group A (33/84, 39.3%) and group B (20/114, 17.5%) was significant (*P* = 0.001).

Multivariate logistic regression analysis showed that a favorable outcome after 1 year was associated with the difference in therapies, age, GCS, HV, IVH and pulmonary infection (all *P* <0.05).

**Conclusions:**

For patients with hypertensive spontaneous ICH (HV≧30 mL in basal ganglia), MIPD may be a more effective treatment than DC, as assessed by a higher rate of functional independence at 1 year after onset as well as reduced mortality in patients ≦60 years of age, NIHSS < 15 or HV≦60 mL.

## Background

Hypertensive intracranial hemorrhage (ICH) in the area of the basal ganglia accounts for 50-70% of all spontaneous ICH, and mortality at 30 days after onset is 33.3% to 50.6%, [1, 2] while 41% of survivors has some degree of disability [3]. Hemostasis and clot removal achieved pathophysiologic benefits, including the prevention of hemorrhagic expansion, reduction of intracranial pressure and clot mass effect [4]. However, the optimal treatment choice for ICH, medical or surgical, continues to be controversial.

The largest prospective randomized study, the STICH trial [5], with 1033 patients from 107 centers over an 8-year period, indicated that surgical evacuation did not appear to be helpful in treating supratentorial ICH. The STICH II trial [6] was recently completed using including 601 conscious patients with superficial lobar intracerebral hemorrhages (10–100 mL) and without intraventricular hemorrhage, who received either early surgery or conservative treatment in a ratio of 1:1. This trial showed no significant outcome differences at 6 moths between the two groups.

Surgical hematoma evacuation failed to demonstrate a benefit to survival or morbidity, which may be attributable to some additional damage incurred to uninjured brain overlying the hematoma caused by the surgical approach. To minimize this risk, minimally invasive surgical strategies have been used.

A randomized controlled study by Miller et al. [7] investigated 10 patients with ICH who received endoscopic aspiration or a conservative treatment and found lower 6 month mortality in the endoscopic group than the medical group. However, the efficacy in this very small trial was limited to superficial lobe hematomas. Nishihara et al. [8] found that endoscopic evacuation provided better neurological outcomes than CT-guided stereotactic hematoma removal. However, among their 27 cases in the endoscopic group, more than 50% of the 15 patients with a good outcome had a small subcortical or cerebellar hemorrhage. The general outcomes of endoscopic and conservative treatments for basal ganglia hemorrhages have been shown to have no significant difference between them [9]. Thus, there is insufficient evidence to confirm that these treatments can improve functional outcomes of ICH.

The optimal approach to remove a hematoma resulting from ICH would be a rapid, simple method that combines a high success rate with low risk and minimal cost. One technique that may have such characteristics is minimally invasive puncture and drainage (MIPD). MIPD is widely used in China; however, few studies have compared the efficacy of MIPD and DC for patients with hemorrhages of 30 mL or more in the basal ganglia. The present study was designed to investigate whether MIPD could improve outcomes in these patients compared with the traditional decompressive craniectomy (DC).

## Methods

### Inclusion criteria

Patients were eligible for the study if they had a hypertensive spontaneous ICH in the basal ganglia with a hematoma volume (HV) ≧30 mL, the hematoma evacuation could start within 24 hours of ictus (if the onset was unobserved, it was considered to be at the last time the patient was definitely normal), and the informed consent for the operation could be obtained from patient’s relative or guardian.

### Exclusion criteria

Patients with ICH located in the cerebral lobes, infratentorial or subarachnoid areas of the brain; ICH caused by trauma, aneurysms, arteriovenous malformation; ICH secondary to an ischemic infarction or coagulopathy; or patients with previous neurological defects or without definite hypertension were excluded.

### Patients

Consecutive patients with hypertensive spontaneous ICH were non-randomly admitted to General Hospital of the Beijing Military Region and Xianghe Hospital from February 2009 to February 2012. The study was approved by the Ethics Committee of General Hospital of Beijing Military Region.

### Grouping

Patients who presented to the General Hospital of the Beijing Military Region were non-randomly admitted by an emergency neurologist to the neurology department to receive MIPD (group A) or to the neurosurgery department to undergo DC (group B) hematoma evacuation. Patients presented to the Xianghe hospital were admitted to its neurology department to receive MIPD. The final assignment of the group for each patient was determined by the patient’s relative, who was presented with the risks and benefits of each ICH treatment from the neurologist: DC (general anesthesia, craniectomy, thorough hematoma evacuation under direct vision), MIPD (local anesthesia, minimal invasion, non direct vision, continuous drainage) or conservative medical treatment. All MIPD procedures in both hospitals were performed by the first author.

### Baseline index definition and classification

Hypertension was judged to be present if the patient fulfilled one of the following criteria: (1) treatment with antihypertensive drugs, previously or currently; (2) repeated measurements of systolic blood pressure >160 mmHg or diastolic blood pressure >95 mmHg after admission. Diabetes mellitus (DM) was assessed if the patient had a history of DM or was taking anti-DM medications.

The scores from the Glasgow Coma Scale (GCS) were classified into 3 categories: mild (15–13), moderate (12–7) and severe (6–3). Scores from the National Institutes of Health Stroke Scale (NIHSS) were similarly classified: mild (<15), moderate (15–20) and severe (>20).

ICH was diagnosed using a CT scan, with the hematoma located in the basal ganglia (internal and/or external capsule, caudate nucleus, thalamus, putamen, or more than one of the above structures). HV was calculated from the CT using the formula ABC/2, [10] where “A” and “B” represent the length and width diameters, respectively, of the largest hemorrhage slice, and “C” is the slice thickness, in centimeters. We classified HV into 2 categories: ≦60 mL and >60 mL. The degree of an extended intraventricular hemorrhage (IVH) was classified into 4 categories, using the methods described by Graeb [11]: 0, 1–4, 5–8 and 9–12 (from none to severe).

Time from onset to operation and complications, including rebleed (RB), renal failure (RF), pulmonary infection (PI) and upper gastrointestinal bleed (UGB) were also recorded.

### Interventions

#### Basal treatment

All patients received basal medical management, including treatments for the control of cerebral edema, blood pressure and glycemia; gastric cytoprotection; nutritional support; and the prevention of complications.

#### DC

Patients in group B received DC. After approximately 10*10 cm^2^ temporoparietal craniectomy, the dura was opened in a cruciate manner; the hematoma was removed microscopically with a suction device through the middle temporal gyrus. Active bleeding was controlled with standard neurosurgical techniques. An external ventricular drain tube was set into the lateral ventricle for drainage, if IVH was present. The scalp was then closed.

#### MIPD

**Figure 1:**
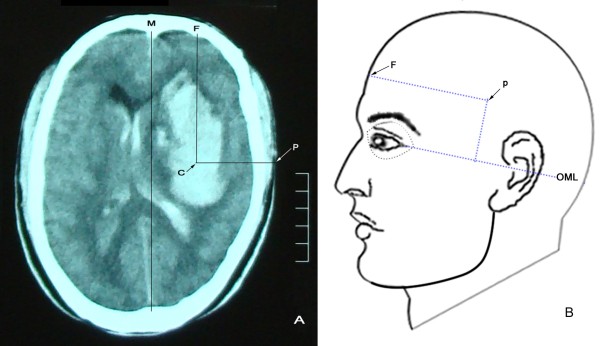
**Brain CT scan slice with the maximum hematoma area above the outer canthus-meatus line (OML) with the puncture point shown. A**: brain CT. Extension lines from the center (C) of the hematoma vertically and horizontally outward to the frontal (F) scalp and temporal puncture (P) point are represented by CF and CP, respectively. Here, CF is parallel to the median sagittal line (M), and CP is perpendicular to CF. P is the puncture point. Puncture depth is the distance between C and P. **B**: a diagram of the puncture point on the body surface. Here, the distance from F to P is equal to the CF line and parallel to the OML. The vertical length from the OML to P on the body surface is equal to the height (mm) from the OML to the maximum hematoma slice on the CT film.

Patients in group A underwent MIPD as follows: 1) The body surface puncture site of the hematoma was determined based on the maximum hematoma slice on the CT film (Figure 1), avoiding major vessels and important functional areas. 2) A YL-I puncture needle (Beijing WanTeFu Medical Apparatus Co., Ltd. http://www.bjwtf.com/en) was used; the length (mm) of the selected needle matched with the depth from the temporal scalp to the hematoma center. 3) After local anesthesia, the needle was drilled into the center of the hematoma via the surface localization puncture point, perpendicularly to the sagittal plane. 4) The drill bit of the needle was removed and a drainage tube was connected to the side hole of the needle. A 5-mL syringe was connected to the other end of the tube, and uncoagulated blood was gently aspirated. 5) The hematoma cavity was rinsed with saline through an ancillary washing needle. 6) Urokinase (10000 U)/saline (3 mL) was infused into the clot, which was bathed for 1 hour and then drained into a closed collection bag. 7) A follow-up CT scan was obtained 12–24 hours after MIPD. Step 6 was repeated if residual blood remained, until the hemorrhage was completely removed or until the remaining HV was less than 10 mL (2–7 days were generally required). Subsequently, the puncture needle was removed and the puncture site was bandaged for 5–7 days. If severe IVH was present, lateral ventricular external drainage was performed just after step 6.

#### Follow-up

The primary outcome was death at 30 days after onset. Functional independence was assessed at 1 year using the Glasgow Outcome Scale [12] (GOS, scores range from 1 to 5, score 1 indicating death, ≥4 indicating functional independence, with lower scores indicating greater disability). The follow-up was completed by observer-blind neurologists via telephone or via an interview with the patient or their relative.

#### Statistics

Measurement data were expressed as “” when the data were distributed normally; otherwise, the data were expressed as “M (Q_1_ ~ Q_3_)”. Baseline grouped measurement data that met the normality and homogeneity of variances criteria were analyzed using Student’s *t* test; otherwise, the Wilcoxon rank sum test was used. Chi-square or rank sum tests were used for categorical data. Because the outcome variable was ordinal, the Wilcoxon rank sum test was used to compare the difference between groups. Prognostic factors were analyzed using binary logistic regression (forward conditional method). For patients lost during follow-up, the patients’ last observation was considered as the final outcome status to be included in the intention-to-treat analysis. All analyses were performed with SPSS 18.0. *P* <0.05 was considered to be a statistically significant difference.

## Results

### General result

**Figure 2:**
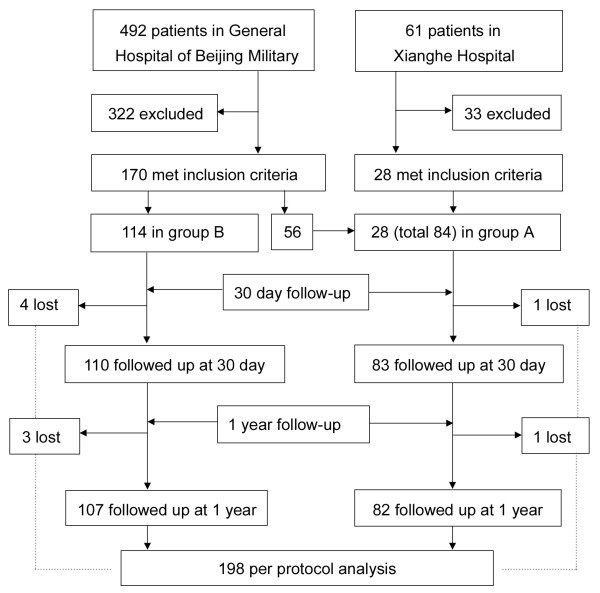
**Trial profile.** A total of 553 consecutive patients with ICH were admitted. Of these patients, 492 were admitted to the General Hospital of Beijing Military Region and 61 were admitted to the Xianghe Hospital. Based on the inclusion/exclusion criteria, 355 were excluded, and 198 were available according to the per protocol sample. Of those, 84 received minimally invasive puncture and drainage (group A), and 114 underwent decompressive craniectomy (group B) hematoma evacuations. Nine were lost during follow up at 30 days (1 and 4) and 1 year (1 and 3 in group A and group B, respectively). For those patients, their last observed data were used as their final results for intention-to-treat analysis.

Baseline data are shown in Table 1 and Figure 2. A total of 553 consecutive patients with spontaneous ICH in the basal ganglia were admitted. Of these patients, 355 were excluded (most for having HV < 30 mL, refusing the operation, or having additional complications). Therefore, 198 patients were available for this study, including 9 cases that were lost during follow up. There were 84 cases in group A and 114 cases in group B, of which 2 and 7 were lost in each group, respectively, during follow up. The mean age was 57.1 ± 12.8 years (ranged 31–95), and the age of group A was older than group B (59.4 ± 14.5 vs. 55.3 ± 11.1 years, *P =* 0.025). The mean HV was 56.7 ± 23.0 mL (ranged 10–144 mL). Patients with IVH accounted for 67.7% of the total patient population used. Except for age, there were no other significant differences between the two groups in their baseline data, including gender, blood pressure, initial GCS, NIHSS and the time from ictus to operation.

**Table 1:** **Clinic baseline characteristics**

Characteristics	All patients (n = 198)	Groups		***P***
		A (n = 84)	B (n = 114)	
Age, mean ± SD (range)	57.1 ± 12.8 (31–95)	59.4 ± 14.5 (31–95)	55.3 ± 11.1 (36–87)	0.025^*a*^
≦60, n (%)	49.5 ± 6.7, 130 (65.7)	48.4 ± 7.2, 45 (53.6)	50.0 ± 6.4, 85 (74.6)	0.209^*a*^
> 60, n (%)	71.5 ± 8.3, 68 (34.3)	72.1 ± 9.5, 39 (46.4)	70.9 ± 6.4, 29 (25.4)	0.562^*a*^
Female/Male, n (%)	54 (27.3)/144 (72.7)	22 (26.2)/62 (73.8)	32 (28.1)/82 (71.9)	0.769
Diabetes, No/Yes, n (%)	127 (64.1)/71 (35.9)	60 (71.4)/24 (28.6)	67 (58.8)/47 (41.2)	0.066
Time from ictus to operation, h				0.123^*b*^
Median (25%, 75%)	7 (5, 9)	7 (6, 10)	6 (5, 9)	
BP (mm Hg), mean ± SD				
SBP	188.1 ± 26.4	185.9 ± 24.5	189.8 ± 27.7	0.300^*a*^
DBP	103.4 ± 12.1	102.1 ± 11.1	104.3 ± 12.7	0.108^*a*^
GCS, mean ± SD	8.1 ± 3.4	8.6 ± 3.6	7.8 ± 3.2	0.082
15-13, n (%)	30 (15.2)	17 (20.2)	13 (11.4)	0.111^*b*^
12-7, n (%)	88 (44.4)	37 (44.0)	51 (44.7)	
6-3, n (%)	80 (40.4)	30 (35.7)	50 (43.9)	
NIHSS, mean ± SD	20.8 ± 5.3	20.2 ± 5.5	21.2 ± 5.1	0.194
<15, n (%)	32 (16.2)	17 (20.2)	15 (13.2)	0.163 ^*b*^
15-20, n (%)	50 (25.3)	22 (26.2)	28 (24.6)	
>20, n (%)	116 (58.6)	45 (53.6)	71 (62.3)	
HV, mL (Range)	56.7 ± 23.0 ( 30–144)	53.7 ± 23.4 (30–144)	58.9 ± 22.5 (30–128)	0.119^*a*^
≦60 mL, n (%)	43.12 ± 8.9, 131 (66.2)	41.9 ± 8.9, 61 (72.6)	44.2 ± 8.8, 70 (61.4)	0.142^*a*^
> 60 mL, n (%)	83.2 ± 18.6, 67 (33.8)	85.0 ± 21.1, 23 (27.4)	82.2 ± 17.3, 44 (38.6)	0.560 ^*a*^
IVH, n (%),				0.219^*b*^
0	64 (32.3)	30 (35.7)	34 (29.8)	
1-4	62 (31.3)	27 (32.1)	35 (30.7)	
5-8	36 (18.2)	15 (17.9)	21 (18.4)	
9-12	36 (18.2)	12 (14.3)	24 (21.1)	

*P* values were calculated using the Chi-square , Student’s *t* (^*a*^) or Wilcoxon rank sum tests (^*b*^). Groups: A, minimal invasive puncture and drainage; B, decompressive craniectomy; BP: blood pressure; SPB systolic blood pressure. DBP: diastolic blood pressure. GCS: Glasgow Coma Scale; NIHSS: National Institutes of Health Stroke Scale; HV: hematoma volume; IVH: intraventricular hemorrhage.

### Short-term outcome

Table 2 shows the outcomes at 30 days after ictus. The cumulative total mortality was 32.3% (64/198), and the difference between group A (27.4%) and group B (36.0%) was not statistically significant (OR = 1.490, 95% CI 0.807 ~ 2.751, *P =* 0.203). Additionally, no significant difference was observed between the two groups in the incidence of complications (RB, PI, RF and UGB) and their corresponding mortality. In addition, the incidence of secondary intracranial infection (0 and 2 in groups A and B, respectively) and required reoperation (0 and 2 in groups A and B, respectively) was indistinguishable between the groups.

**Table 2:** **Mortality at 30 days**

	All Patients n (%)			Groups						
				A, n = 84 (%)			B, n = 114 (%)			
	Dead (GOS = 1)	Survival GOS > 1)	P	Dead (GOS = 1)	Survival GOS > 1)	Total	Dead (GOS = 1)	Survival (GOS > 1)	Total	***P***
Total	64 (32.3)	134 (67.7)		23 (27.4)	61 (72.6)	84	41 (36.0)	73 (64.0)	114	0.203
Age										0.203
≦60	42 (32.3)	88 (67.7)	0.995	9 (20.0)	36 (80.0)	45	33 (38.8)	52 (61.2)	85	0.030
> 60	22 (32.4)	46 (67.6)		14 (35.9)	25 (64.1)	39	8 (27.6)	21 (72.4)	29	0.472
Gender										0.203
Female	14 (25.9)	40 (74.1)	0.240	4 (18.2)	18 (81.8)	22	10 (31.3)	22 (68.8)	32	0.286
Male	50 (34.7)	94 (65.3)		19 (30.6)	43 (69.4)	62	31 (37.8)	51 (62.2)	82	0.373
Diabetes										0.203
No	34 (26.8)	93 (73.2)	0.026	12 (20.0)	48 (80.0)	60	22 (32.8)	45 (67.2)	67	0.104
Yes	30 (42.3)	41 (57.7)		11 (45.8)	13 (54.2)	24	19 (40.4)	28 (59.6)	47	0.665
GCS			<0.001							0221
15-13 grade 1	2 (6.7)	28 (93.3)		0 (0.0)	17 (100.0)	17	2 (15.4)	11 (84.6)	13	0.179
12-7 grade 2	21 (23.9)	67 (76.1)	0.059^*a*^	7 (18.9)	30 (81.1)	37	14 (27.5)	37 (72.5)	51	0.357
6-3 grade 3	41 (51.3)	39 (48.8)	<0.001^*b*^	16 (53.3)	14 (46.7)	30	25 (50.0)	25 (50.0)	50	0.477
NIHSS			0.001							0.221
<15 grade 1	5 (15.6)	27 (84.4)		0 (0.0)	17 (100)	17	5 (33.3)	10 (66.7)	15	0.015
15-20 grade 2	9 (18.0)	41 (82.0)	1.000.^*a*^	3 (13.6)	19 (86.4)	22	6 (21.4)	22 (78.6)	28	0.713
>20 grade 3	50 (43.1)	66 (56.9)	0.002^*b*^	20 (44.4)	25 (55.6)	45	30 (42.3)	41 (55.7)	71	0.817
HV, mL										0.203
≦60	29 (22.1)	102 (77.9)	<0.001	8 (13.1)	53 (86.9)	61	21 (30.0)	49 (70.0)	70	0.021
> 60	35 (52.5)	32 (47.8)		15 (65.2)	8 (34.8)	23	20 (45.5)	24 (54.5)	44	0.127
IVH			<0.001							0.221
0	12 (18.8)	52 (81.3)		3 (10.0)	27 (90.0)	30	9 (26.5)	25 (73.5)	34	0.117
1-4 grade 1	10 (16.1)	52 (83.9)	0.816^*c*^	3 (11.1)	24 (88.9)	27	7 (20.0)	28 (80.0)	35	0.491
5-8 grade 2	19 (52.8)	17 (47.2)	<0.001^*a*^	6 (40.0)	9 (60.0)	15	13 (61.9)	8 (38.1)	21	0.201
9-12 grade 3	23 (63.9)	13 (36.1)	0.471^*b*^	11 (91.7)	1 (8.3)	12	12 (50.0)	12 (50.0)	24	0.025
Complications, n (%)										
RB										0.221
No	57 (30.2)	132 (69.8)	0.006	22 (26.5)	61 (73.5)	83	35 (33.0)	71 (67.0)	106	0.334
Yes	7 (77.8)	2 (22.2)		1 (100)	0 (0.0)	1	6 (75.0)	2 (25.0)	8	1.000
RF										0.221
NO	50 (28.2)	127 (71.8)	0.001	19 (24.1)	60 (75.9)	79	31 (31.6)	67 (68.4)	98	0.267
Yes	14 (66.7)	7 (33.3)		4 (80.0)	1 (20.0)	5	10 (62.5)	6 (37.5)	16	0.624
PI										0.203
No	31 (26.7)	85 (73.3)	0.046	12 (24.0)	38 (76.0)	50	19 (28.8)	47 (71.2)	66	0.566
Yes	33 (40.2)	49 (59.8)		11 (32.4)	23 (67.6)	34	22 (45.8)	26 (54.2)	48	0.223
UGB										0.221
No	56 (30.6)	127 (69.4)	0.087	19 (24.7)	58 (75.3)	77	37 (34.9)	69 (65.1)	106	0.139
Yes	8 (53.3)	7 (46.7)		4 (57.1)	3 (42.9)	7	4 (50.0)	4 (50.0)	8	1.000

*P* values were calculated using the Wilcoxon or Kruskal-Wallis rank sum tests. Groups: A, minimal invasive puncture and drainage; B, decompressive craniectomy; GOS: Glasgow Outcome Scale; BP: blood pressure; SPB systolic blood pressure. DBP: diastolic blood pressure. GCS: Glasgow Coma Scale; NIHSS: National Institutes of Health Stroke Scale; HV: hematoma volume; IVH: intraventricular hemorrhage; RB: rebleed; PI: pulmonary infection; RF: renal failure; UGB: upper gastrointestinal bleeding; ^*a*^grade 1 vs. grade 2; ^*b*^grade 2 vs. grade 3; ^*c*^IVH 0 vs. IVH grade 1.

Further analysis using stratified variables indicated that for patients ≦60 years of age, NIHSS <15, or HV≦60 mL, the mortality of group A was significantly lower than that of group B. For patients with severe IVH, the mortality of group B was significantly lower than that of group A.

Univariate analysis showed that for patients with DM, GCS≦ 6, NIHSS > 20, HV > 60 mL or IVH >4 or with complications (RB, RF or PI), the risk of mortality at 30 days after ictus was significantly increased.

Logistic regression analysis (Table 3) using the raw data revealed that after adjusting for age, sex and other factors, independent predictors of 30-day mortality included a lower baseline GCS and the presence of IVH combined with RB and RF. For a patient with RB or RF, the risk of death increased to more than 13 or 3 times, respectively.

**Table 3:** **Binary logistic analyze of morality risk at 30-day of ictus**

Clinical factors	B	S.E.	Wald	df	Sig.	Exp(B)	95% CI	
							Lower	Upper
GCS	−0.162	0.065	6.156	1	0.013	0.850	0.748	0.967
IVH	0.194	0.049	15.818	1	0.000	1.214	1.103	1.335
RB	2.574	0.890	8.354	1	0.004	13.113	2.290	75.099
RF	1.227	0.566	4.706	1	0.030	3.410	1.126	10.331
Constant	−3.184	0.587	29.451	1	<.001	0.041		

Method: Forward conditional; Entry 0.05, Removal: 0.10, Classification cutoff: 0.5. Dependent: outcome at 30-day of ictus: 0 = survival, 1 = dead. IVH: intraventricular hemorrhage; RB: rebleed, 0 = no, 1 = yes; RF: renal failure, 0 = no, 1 = yes. All covariates used were original data.

The cumulative total favorable outcome (GOS > 3) at 30 days post onset was 10.6% (21/198), i.e., only 21 of the 134 survivors were functionally independent. There was no significant difference between group A and group B (11.9%, vs. 11.4%, *P =* 0.914, detailed data not provided).

### Long-term outcome

Table 4 shows that the cumulative total mortality at 1 year after ictus was 43.4% (86/198) and that the difference between group A and group B was not significant (36.1% vs. 48.2%, *P =* 0.112).

**Table 4:** **Morality at 1 year after ICH onset**

	All patients n (%)			Groups						
				A (n = 84)			B (n = 114)			
	Dead	Alive	***P***	Dead	Alive	Total	Dead	Alive	Total	***P***
	86 (43.4)	112 (56.6)		31 (36.1)	53 (63.9)	84	55 (48.2)	59 (51.8)	114	0.112
Age										0.112
≦60	50 (38.5)	80 (61.5)	0.052	11 (24.4)	34 (75.6)	45	39 (45.9)	46 (51.4)	85	0.017
> 60	36 (52.9)	32 (47.1)		20 (51.3)	19 (48.7)	39	16 (55.2)	19 (48.7)	39	0.752
Gender										0.112
Female	22 (40.7)	32 (59.3)	0.640	9 (40.9)	13 (59.1)	22	13 (40.6)	19 (59.4)	32	0.983
Male	64 (44.4)	80 (55.6)		22 (35.5)	40 (64.5)	62	42 (51.2)	40 (48.8)	82	0.061
Diabetes										0.112
No	48 (37.8)	79 (62.2)	0.033	18 (30.0)	42 (70.0)	60	30 (44.8)	37 (55.2)	67	0.088
Yes	38 (53.5)	33 (46.5)		13 (54.2)	11 (45.8)	24	25 (53.2)	22 (46.8)	47	0.938
GCS			<0.001							0.147
15-13 grade 1	3 (10.0)	27 (90.0)		0 (0.0)	17 (100.0)	17	3 (23.1)	10 (76.9)	13	0.070
12-7 grade 2	29 (33.0)	59 (67.0)	0.017^*a*^	8 (21.6)	29 (78.4)	37	21 (41.2)	30 (58.8)	51	0.055
6-3 grade 3	54 (67.5)	26 (32.5)	<0.001^*b*^	23 (76.7)	7 (23.3)	30	31 (62.0)	19 (38.0)	50	0.178
NIHSS			<0.001							0.147
<15 grade 1	5 (15.6)	27 (84.4)		0 (0.0)	17 (100.0)	17	5 (33.3)	10 (66.7)	15	0.015
15-20 grade 2	14 (28.0)	36 (72.0)	0.284^*a*^	4 (18.2)	18 (81.8)	22	10 (35.7)	18 (64.3)	28	0.175
>20 grade 3	67 (57.8)	49 (42.2)	<0.001^*b*^	27 (60.0)	18 (40.0)	45	40 (56.3)	31 (43.7)	71	0.698
HV, mL										0.112
≦60	41 (31.3)	90 (68.7)	<0.001	13 (21.3)	48 (78.7)	61	28 (40.0)	42 (60.0)	70	0.022
> 60	45 (67.2)	22 (32.8)		18 (78.3)	5 (21.7)	23	27 (61.4)	17 (38.6)	44	0.165
IVH			<0.001							0.147
0	16 (25.0)	48 (75.0)	0.918^c^	3 (10.0)	27 (90.0)	30	13 (38.2)	21 (61.8)	34	0.010
1-4	16 (25.8)	46 (74.2)		5 (18.5)	22 (81.5)	27	11 (31.4)	24 (68.6)	35	0.253
5-8	27 (75.0)	9 (25.0)	<0.001^*a*^	11 (73.3)	4 (26.7)	15	16 (76.2)	5 (23.8)	21	1.000
9-12	27 (75.0)	9 (25.0)	1.000^*b*^	12 (100.0)	0 (0.0)	12	15 (62.5)	9 (37.5)	24	0.036
Complications, n (%)										
RB										0.147
No	79 (41.8)	110 (58.2)	0.042	30 (36.1)	53 (63.9)	83	49 (46.2)	57 (53.8)	106	0.164
Yes	7 (77.8)	2 (22.2)		1 (100.0)	0	1	6 (75.0)	2 (25.0)	8	1.000
RF										0.147
NO	68 (38.4)	109 (61.6)	<0.001	26 (32.9)	53 (67.1)	79	42 (42.9)	56 (57.1)	98	0.177
Yes	18 (85.7)	3 (14.3)		5 (100.0)	0	5	13 (81.3)	3 (18.8)	16	0.549
PI										0.147
No	38 (32.8)	78 (67.2)	<0.001	13 (26.0)	37 (74.0)	50	25 (37.9)	41 (62.1)	66	0.179
Yes	48 (58.5)	34 (41.5)		18 (52.9)	16 (47.1)	34	30 (62.5)	18 (37.5)	48	0.496
UGB										0.147
No	75 (41.0)	108 (59.0)	0.027	25 (32.5)	52 (67.5)	77	50 (47.2)	56 (52.8)	106	0.046
Yes	11 (73.3)	4 (26.7)		6 (85.7)	1 (14.3)	7	5 (62.5)	3 (37.5)	8	0.569

*P* values were calculated using the Wilcoxon or Kruskal-Wallis rank sum tests. Groups: A, minimal invasive puncture and drainage; B, decompressive craniectomy; BP: blood pressure; SPB systolic blood pressure. DBP: diastolic blood pressure. GCS: Glasgow Coma Scale; NIHSS: National Institutes of Health Stroke Scale; HV: hematoma volume; IVH: intraventricular hemorrhage; RB: rebleed; PI: pulmonary infection; RF: renal failure; UGB: upper gastrointestinal bleeding; ^*a*^grade 1 vs. grade 2; ^*b*^grade 2 vs. grade 3; ^*c*^IVH 0 vs. IVH grade 1.

Further analysis using stratified variables showed that for patients ≦60 years of age, NIHSS <15, HV≦60 mL, no IVH, or no UGB, the 1-year mortality of group A was significantly lower than that for group B. For patients with IVH scores of 9–12, the 1-year mortality of group B was obviously lower than that of group A. Other stratified variables showed no significant differences between groups.

Using univariate analysis, higher mortality was shown to be closely related with DM (53.5%), initial GCS≦6 (67.5%), NIHSS > 20 (57.8%), HV > 60 mL (67.2%) and IVH > 4 (75%) and also with any complication, RB (77.8%), RF (85.7%), PI (58.5%) and UGB (73.3%).

Using multivariate analysis (Table 5) on the raw data after being adjusted for other factors, age, GCS, HV, IVH, RB and RF were determined to be independent risk factors for fatality at 1 year: the higher the initial GCS score, the lower the risk of death at 1 year after onset. If a patient had RB or RF complications, the risk of death at 1 year was increased by 8 or 6 times, respectively.

**Table 5:** **Binary logistic analysis of morality risks at 1 year post ictus**

Clinical factors	B	S.E.	Wald	df	Sig.	Exp(B)	95% CI	
							Lower	Upper
Age	0.029	0.014	4.185	1	0.041	1.030	1.001	1.059
GCS	−0.172	0.066	6.782	1	0.009	0.842	0.739	0.958
HV	0.021	0.010	4.220	1	0.040	1.021	1.001	1.042
IVH	0.182	0.051	12.588	1	0.000	1.200	1.085	1.327
RB	2.170	0.935	5.389	1	0.020	8.758	1.402	54.706
RF	1.881	0.730	6.636	1	0.010	6.561	1.568	27.448
Constant	−2.752	1.172	5.512	1	0.019	0.064		

Method: Forward conditional; Entry 0.05, Removal: 0.10, Classification cutoff: 0.5. Dependent: outcome at 1-year of ictus: 0 = survival, 1 = dead. GCS: Glasgow Coma Scale; HV: hemorrhagic volume; IVH: intraventricular hemorrhage; RB: rebleed, 0 = no, 1 = yes; RF: renal failure, 0 = no, 1 = yes; All covariates used were original data.

The 1-year prognosis-based GOS showed favorable outcomes in 39.3% of the patients in group A and in 17.5% of patients in group B (*P =* 0.001, Table 6); therefore, the absolute difference in the favorable outcome between MIPD and DC was 21.8% (OR = 0.329, 95% CI 0.171 to 0.631, *P =* 0.001). Logistic regression analysis indicated that different treatments, age, GCS, HV, IVH and PI were all significant factors for favorable outcomes at 1 year (Table 7). Specifically, MIPD treatment, younger age, higher initial GCS, less HV and the absence of IVH/PI were associated with more favorable outcomes at 1 year after ictus.

**Table 6:** **Comparison of good outcome at 1 year**

	All Patients n (%)			Groups						
				A (n = 84)			B (n = 114)			
	Bad (GOS≦3)	Good (GOS > 3)	***P***	Bad (GOS≦3)	Good (GOS > 3)	Total	Bad (GOS≦3)	Good (GOS > 3)	Total	***P***
	145 (73.2)	53 (26.8)		51 (60.7)	33 (39.3)	84	94 (82.5)	20 (17.5)	114	0.001
Age										0.001
≦60	85 (65.4)	45 (34.6)	0.001	18 (40.0)	27 (60.0)	45	67 (78.8)	18 (21.2)	85	<0.001
> 60	60 (88.2)	8 (11.8)		33 (84.6)	6 (15.4)	39	27 (93.1)	2 (6.9)	29	0.451
Gender										0.001
Female	40 (74.1)	14 (25.9)	0.870	15 (68.2)	7 (31.8)	22	25 (78.1)	7 (21.9)	32	0.417
Male	105 (72.9)	39 (27.1)		36 (58.1)	26 (41.9)	62	69 (84.1)	13 (15.9)	82	0.001
Diabetes										0.001
No	85 (66.9)	42 (33.1)	0.008	32 (53.3)	28 (46.7)	60	53 (79.1)	14 (20.9)	67	0.002
Yes	60 (84.5)	11 (15.5)		19 (79.2)	5 (20.8)	24	41 (87.2)	6 (12.8)	47	0.490
GCS			<0.001							0.004
15-13 (grade 1)	14 (46.7)	17 (53.3)		5 (29.4)	12 (70.6)	17	9 (69.2)	4 (30.8)	13	0.033
12-7 (grade 2)	55 (62.5)	33 (37.5)	0.130^*a*^	18 (48.6)	19 (51.4)	37	37 (72.5)	14 (27.5)	51	0.023
6-3 (grade 3)	76 (95.0)	4 (5.0)	<0.001^*b*^	28 (93.3)	2 (6.7)	30	48 (96.0)	2 (4.0)	50	0.705
NIHSS			<0.001							0.001
<15 (grade 1)	15 (46.9)	17 (53.1)		4 (23.5)	13 (76.5)	17	11 (73.3)	4 (26.7)	15	0.006
15-20 (grade 2)	28 (56.0)	22 (44.0)	0.422^*a*^	10 (45.5)	12 (54.5)	22	18 (64.3)	10 (35.7)	28	0.187
>20 (grade 3)	102 (87.9)	14 (12.1)	<0.001^*b*^	37 (82.2)	8 (17.8)	45	65 (91.5)	6 (8.5)	71	0.135
HV , mL										0.001
≦60	80 (61.1)	51 (38.9)	<0.001	29 (47.5)	32 (52.5)	61	51 (72.9)	19 (27.1)	70	0.003
> 60	65 (97.0)	2 (3.0)		22 (95.7)	1 (4.3)	23	43 (97.7)	1 (2.3)	44	1.000
IVH			<0.001							0.001
0	31 (48.4)	32 (51.6)		10 (33.3)	20 (66.7)	30	21 (61.8)	13 (38.2)	34	0.024
1-4 (grade 1)	43 (69.4)	19 (30.6)	0.018^c^	14 (51.9)	13 (48.1)	27	29 (82.9)	6(17.1)	35	0.009
5-8 (grade 2)	36 (100.0)	0	<0.001^*a*^	15 (100.0)	0	15	21 (100.0)	0	21	1.000
9-12 (grade 3)	35 (97.2)	1 (2.8)	1.000	12 (100.0)	0	12	23 (95.8)	1 (4.2)	24	1.000
Complications, n (%)										
RB										0.001
No	136 (72.0)	53 (28.0)	0.116	50 (60.2)	33 (39.8)	83	86 (81.1)	20 (18.9)	106	0.002
Yes	9 (100.0)	0		1 (100.0)	0	1	8 (100)	0	8	1.000
RF										0.001
NO	124 (70.1)	53 (29.9)	0.003	46 (58.2)	33 (41.8)	79	78 (79.6)	20 (20.4)	98	0.002
Yes	21 (100.0)	0		5 (100.0)	0	5	16 (100.0)	0	16	1.000
PI										0.001
No	70 (60.3)	46 (39.7)	<0.001	22 (44.0)	28 (56.0)	50	48 (72.7)	18 (27.3)	66	0.002
Yes	75 (91.5)	7 (8.5)		29 (85.3)	5 (14.7)	34	46 (95.8)	2 (4.2)	48	0.120
UGB										0.001
No	131 (71.6)	52 (28.4)	0.075	44 (57.1)	33 (42.9)	77	87 (82.1)	19 (17.9)	106	<0.001
Yes	14 (93.3)	1 (6.7)		7 (100.0)	0	7	7 (87.5)	1 (12.5)	8	1.000

*P* values were calculated using the Wilcoxon or Kruskal-Wallis rank sum tests. Groups: A, minimal invasive puncture and drainage; B, decompressive craniectomy; GOS: Glasgow Outcome Scale; BP: blood pressure; SPB systolic blood pressure. DBP: diastolic blood pressure. GCS: Glasgow Coma Scale; NIHSS: National Institutes of Health Stroke Scale; HV: hematoma volume; IVH: intraventricular hemorrhage; RB: rebleed; PI: pulmonary infection; RF: renal failure; UGB: upper gastrointestinal bleeding; ^*a*^grade 1 vs. grade 2; ^*b*^grade 2 vs. grade 3; ^*c*^IVH 0 vs. IVH grade 1.

**Table 7:** **Binary logistic regression analyze of good outcome factors at 1 year**

Variables	B	S.E.	Wald	df	Sig.	Exp(B)	95% CI	
							Lower	Upper
Group	−1.274	0.504	6.381	1	0.012	0.280	0.104	0.752
Age	−1.538	0.581	7.004	1	0.008	0.215	0.069	0.671
GCS	0.171	0.082	4.352	1	0.037	1.187	1.010	1.395
HV	−0.059	0.021	8.065	1	0.005	0.943	0.906	0.982
IVH	−0.423	0.132	10.268	1	0.001	0.655	0.506	0.849
PI	−1.556	0.554	7.900	1	0.005	0.211	0.071	0.624
Constant	5.503	1.717	10.276	1	0.001	245.452		

Method: Forward conditional; Entry 0.05, Removal: 0.10, Classification cutoff: 0.5. Dependent: outcome at 1-year of ictus: 0 = bad, 1 = favorable (Glasgow Outcome Scale score≧3); Group: 1 = minimal invasive puncture and drainage, 2 = decompressive craniectomy; GCS: Glasgow Coma Scale; HV: hemorrhagic volume; IVH: intraventricular hemorrhage; PI: pulmonary infection, 0 = no, 1 = yes. All covariates used were original data except for Age (1, ≦60 years; 2, >60 years).

## Discussion

The mass effect of a hematoma can lead to brain damage, such as intracranial hypertension or a cerebral hernia. Some evidence suggests that the mass effect caused by HV (<60 mL) is not the dominant injury mechanism; rather, excitotoxic substances released from the hematoma, such as elevated glutamate levels in the perihematomal region, may have an important impact on secondary cerebral injury [13]. Therefore, the effective removal of the hematoma at the acute phase is crucial for the effective treatment of ICH and would allow for reducing mortality and improving long-term quality of life.

Surgical removal of the bone flap is a classical technique for treating ICH; it is characterized by good visibility, complete removal of the hematoma, easy hemostasia, and a reduction of resultant pressure. However, there are also some shortcomings associated with the procedure, such as general anesthesia, duration of the surgery, possible brain distortion, possible damage to the brain tissue around the hematoma by electrocoagulation, rebleeding, and a potential for a series of pathophysiological changes post operation (such as disturbance in water and electrolytes) that can result in the severe impairment of neurological functions and multiple complications.

Several published randomized trials show that there is no benefit from conventional surgery compared with conservative medical treatment; [14, 15] for severe cases, the mortality from DC is as high as 64.7%, and elderly patients rarely survive [16]. The STICH trial [5] failed to demonstrate the assumed superiority of operative treatment over conservative management. The study included 1033 patients (between 19 and 93 years of age; HV between 4 and 210 mL) from 83 participating centers in 27 countries. There was no significant difference in mortality or favorable outcomes between the two groups at 6 months post ictus. Although craniotomy may be helpful in treating patients with lobar clots within 1 cm of the surface that present with GCS > 9, patients in the STICH study with a deep hematoma or with GCS < 8 tended to fare worse with surgical removal compared with medical management.

The recently completed randomized STICH II trial [6] included 601 patients from 78 centers in 27 countries with superficial lobar ICH, a mean age of 65 years, a mean HV of 37 mL (ranged 10–100 mL), and no IVH. The study further indicated that early surgery (99% craniotomy and 1% minimally invasive procedures to evacuate the hematomas within 12 h of ictus) had no significant difference in 6-month mortality (18% and 24%) and good outcome rates (41% and 38%), compared with conservative treatment.

The guidelines for the management of spontaneous ICH by the American Heart Association/American Stroke Association in 2010 [17] recommended that for patients presenting with lobar clots of >30 mL and within 1 cm of the surface, the evacuation of supratentorial ICH by standard craniotomy may be considered; however, for most patients with supratentorial deep ICH, the usefulness of DC is uncertain. An ideal technique for ICH evacuation would be one that minimizes brain manipulation and that could also be performed under minimal anesthesia, preferably at the bedside. The 2007 consensus conference sponsored by the U.S. National Institutes of Health used favorable results of a number of studies and case reports to note that minimally invasive techniques to evacuate clots appear to be a promising area that warrants further investigation [18].

MIPD has become more attractive than DC for treating ICH for the following reasons. First, the YL-1 type puncture needle is designed with a 3.2-mm outer diameter for minimizing the potential exacerbation of secondary brain trauma by avoiding the need for a craniectomy and a brain retraction. Second, the YL-1 type puncture needle has a built-in aiguille for perforating through the skull and dura and has an additional washing needle to replace the aiguille after drilling is complete. For flushing the hematoma, the washing liquid is pushed by a syringe and is ejected from the distal end in all directions, helping to liquefy, dissolve and drain the coagulated clot. Third, the operation can be accomplished at the bedside under local anesthesia in approximately half an hour. For these reasons, it has been widely adopted in China.

However, there are few studies that compare the efficacies of MIPD and DC for deep ICH. The randomized trial by Zhou et al. [19] showed that compared with DC, MIPD did not decrease short-term mortality but significantly improved long-term outcomes.

In the present study, the 30-day mortality rates of groups A and B were similar (27.4% vs. 36.0%, *P =* 0.203); however, good outcomes at 1 year (39.3% vs.17.5% *P <* 0.001) were significantly higher for the MIPD group. Although our MIPD results were similar to those in the STICH II study, all of the patients in STICH II had superficial lobar hemorrhages, absent of IVH, and more than half were fully conscious, whereas all of our patients had hemorrhages at supratentorial deep locations, 67.7% presented with IVH, and 84.8% had a GCS score≦12. The remarkable difference in the favorable long-term outcomes between STICH II and our study suggests that MIPD may be more helpful in improving the neurologic functional outcome of patients with ICH.

Logistic regression analysis revealed that the risk factors for 30-day mortality were GCS, IVH, RB and RF (Table 3). Different treatments, age, GCS, HV, IVH and PI were important factors that impacted the favorable outcomes at 1 year (Table 7).

Age was correlated with ICH prognosis: the older the patients, the higher their risk of death (Table 5); the younger, the greater the likelihood of a favorable outcome (Table 7). For patients ≦60 years of age, both the 30-day and 1-year mortalities of group A were significantly lower, and the 1-year good outcomes were significantly higher than those for group B. For patients older than 60 years of age, there was no noticeable difference in short- or long-term outcomes between the groups. This result implies that MIPD treatment can improve the treatment prognosis for younger patients with ICH.

GCS is the most common scoring system used to describe the level of consciousness and the severity of disability of a patient with brain injury. It correlates well with outcomes following severe brain injury [20]. Our data showed that the 30-day mortality of patients with initial GCS scores of 15–13, 12–7 and 6–3, was 6.7%, 23.9% and 51.3%, respectively; the 1-year mortality was 10.0%, 33.0% and 67.5% and the 1-year favorable outcome was 53.3%, 37.5% and 5.0%, respectively. The 1-year good outcome rates for patients with mild to moderate disturbances of consciousness in group A were more than twice as high as those in group B (70.6% vs. 30.8%, *P =* 0.033; 51.4% vs. 27.5%, *P =* 0.023). This result indicates that MIPD significantly increased the long-term good outcome rate for patients with mild to moderately impaired consciousness.

HV is an important prognosis-associated factor. For patients with HV > 60 mL, the mortality rates at 30 days (52.5%) and 1 year (67.2%) post ictus were two times higher than those with HV < 60 mL (22.1% and 31.3%, respectively, all *P* <0.001), and there was no significant difference between groups A and B. By contrast, for patients with HV≦60 mL, the mortality in group A at 30 days (13.1%) and 1 year (21.3%) was significantly lower than those for group B (30.0% and 40.0%, respectively, all *P <* 0.05). The favorable outcome rate at 1 year was significantly higher in group A than that in group B (52.5% vs. 27.1%, *P =* 0.003). These results suggest that patients with HV≦60 mL may benefit from the treatment of MIPD more than DC, compared with those with HV > 60 mL, in terms of decreasing mortality and increasing neurologic functional independence.

IVH secondary to ICH is a powerful and independent risk factor for poor prognosis, particularly severe IVH. The mortality at 30 days can be as high as 91.7% [1]. The high mortality of IVH is related to the volume of IVH, obstruction of cerebrospinal fluid circulation, [21] and toxic effects of ventricular blood clots, which could lead to secondary cerebral vasospasms and acute obstructive hydrocephalus, especially when the third and fourth ventricles are involved [22].

In the present study, the incidence of IVH was 67.7% (134/198). Of those, 36 cases were severe, and the mortality in group A and group B at 30 days were 91.7% and 50.0% (*P =* 0.025), respectively, and 100% and 62.5% (*P =* 0.036), respectively, at 1 year. DC treatment can be advantageous for reducing mortality in patients with severe IVH. This benefit can be attributed to the more thorough evacuation of the hematoma and more adequate ventricular drainage, which more effectively reduces the obstruction of the circulation of the cerebrospinal fluid. However, the favorable outcome in patients with severe IVH at 1 year was similar between the two groups.

RB is also an independent risk factor for mortality (Table 3, Table 5), with an incidence of 4.5% (9/198, 1 case in group A and 8 cases in group B). Among these, only 2 patients in group B survived at 1-year after ictus but developed serious disabilities. RF is another potentially fatal complication and had an incidence of 10.6% (5 cases in group A and 16 cases in group B). All of the RF cases in group A and 13 of the RF cases in group B died at 1 year post onset; the remaining 3 cases in group B survived with dependency. PI and UGB are also common complications of ICH, with incidences of 41.4% and 7.6%, respectively.

The morbidity rate of the above complications was not significantly different between the two groups. However, in patients without these complications, the long-term good outcome rate was significantly higher in group A than that in group B (all *P* <0.01, Table 6). This result suggests that DC is disadvantageous for neurological function recovery when performed in patients with these complications and that the active prevention and treatment of these complications is essential for improving the prognosis of patients with ICH.

One of the key points of MIPD hematoma evacuation is to ensure that the coagulated clot is completely liquefied, as most acute hematomas are solid or semisolid and may therefore block the drainage system. Thrombolytics have been used to dissolve coagulated clots to make them more amenable to be aspirated or drained.

Trials of minimally invasive surgeries in combination with recombinant tissue plasminogen activator (rtPA) for ICH evacuation show that rtPA, administered both intraventricularly and intracerebrally, are safe and effective for promoting clot dissolution [21, 23]. However, due to its cost, rtPA is not readily available for clot thrombolysis in ICH patients in China. The patients in group A received urokinase injections into the hematoma, with only one case (1.2%) developing RB. Patients in group B did not receive urokinase injections, and 8 cases (7.0%) developed RB, implying that RB may not be associated with urokinase.

## Conclusion

Our results demonstrate that for patients with hypertensive spontaneous ICH (HV≧30 mL in the basal ganglia), MIPD may be more effective than DC for achieving a higher rate of functional independence at 1 year as well as a lower mortality rate in some patients ≦60 years of age, with an NIHSS score <15 or HV≦60 mL. For patient with HV > 60 mL, deep coma or severe IVH, the outcomes of the two therapies were similar. Further randomized control trials are needed to assess the benefits of MIPD for hypertensive spontaneous deep ICH.

## Abbreviations

BP: Blood pressure
CI: Confidence interval
DBP: Diastolic blood pressure
DC: Decompressive craniectomy
DM: Diabetes mellitus
GCS: Glasgow coma scale
ICH: Intracranial hemorrhage
HV: Hematoma volume
IVH: Intraventricular hemorrhage
MIPD: Minimally invasive puncture and drainage
NIHSS: National Institutes of Health Stroke Scale
OR: Odds ratio
PI: Pulmonary infection
RB: Rebleed
RF: Renal failure
rtPA: Recombinant tissue plasminogen activator
SPB: Systolic blood pressure
UGB: Upper gastrointestinal bleeding.
